# Tetra­kis(2,3,5,5-tetra­methyl­hexen-2-yl)silane

**DOI:** 10.1107/S1600536814004322

**Published:** 2014-02-28

**Authors:** Frank Meyer-Wegner, Michael Bolte, Hans-Wolfram Lerner

**Affiliations:** a Institut für Anorganische Chemie, J. W. Goethe-Universität Frankfurt, Max-von-Laue-Strasse 7, 60438 Frankfurt/Main, Germany

## Abstract

In the title compound, C_40_H_76_Si, the Si atom is located on a special position of site symmetry -4. Thus, there is just a quarter of a mol­ecule in the asymmetric unit. The C=C double bonds exhibit a *trans* configuration. The Si atom and the *tert*-butyl group are located on the same side of the plane formed by the C=C double bond and its four substituents. The crystal packing shows no short contacts between the mol­ecules and despite the low crystal density (0.980 Mg m^−3^), there are no significant voids in the structure.

## Related literature   

For information on the chemical background, see: Meyer-Wegner *et al.* (2011 ▶, 2014 ▶).
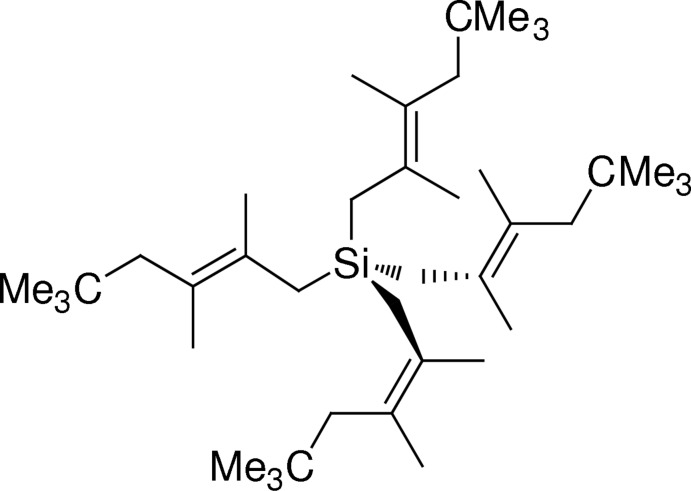


## Experimental   

### 

#### Crystal data  

C_40_H_76_Si
*M* *_r_* = 585.10
Tetragonal, 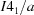

*a* = 12.5780 (11) Å
*c* = 25.053 (3) Å
*V* = 3963.5 (7) Å^3^
*Z* = 4
Mo *K*α radiation
μ = 0.08 mm^−1^
*T* = 173 K
0.32 × 0.28 × 0.16 mm

#### Data collection  

Stoe IPDS-II two-circle diffractometer
Absorption correction: multi-scan (*MULABS*; Spek, 2009 ▶; Blessing, 1995 ▶) *T*_min_ = 0.963, *T*_max_ = 0.988
5117 measured reflections
1742 independent reflections
1301 reflections with *I* > 2σ(*I*)
*R*_int_ = 0.045

#### Refinement  

*R*[*F* ^2^ > 2σ(*F* ^2^)] = 0.051
*wR*(*F* ^2^) = 0.136
*S* = 1.08
1742 reflections
95 parameters
H-atom parameters constrained
Δρ_max_ = 0.25 e Å^−3^
Δρ_min_ = −0.17 e Å^−3^

### 

Data collection: *X-AREA* (Stoe & Cie, 2001 ▶); cell refinement: *X-RED32* (Stoe & Cie, 2001 ▶); data reduction: *X-AREA*; program(s) used to solve structure: *SHELXS97* (Sheldrick, 2008 ▶); program(s) used to refine structure: *SHELXL97* (Sheldrick, 2008 ▶); molecular graphics: *XP* in *SHELXTL* (Sheldrick, 2008 ▶) and *Mercury* (Macrae *et al.*, 2006 ▶); software used to prepare material for publication: *SHELXL97* and *publCIF* (Westrip, 2010 ▶).

## Supplementary Material

Crystal structure: contains datablock(s) I, global. DOI: 10.1107/S1600536814004322/zs2287sup1.cif

Structure factors: contains datablock(s) I. DOI: 10.1107/S1600536814004322/zs2287Isup2.hkl

Supporting information file. DOI: 10.1107/S1600536814004322/zs2287Isup3.cml

CCDC reference: 988679

Additional supporting information:  crystallographic information; 3D view; checkCIF report

## Figures and Tables

**Fig. 1.**
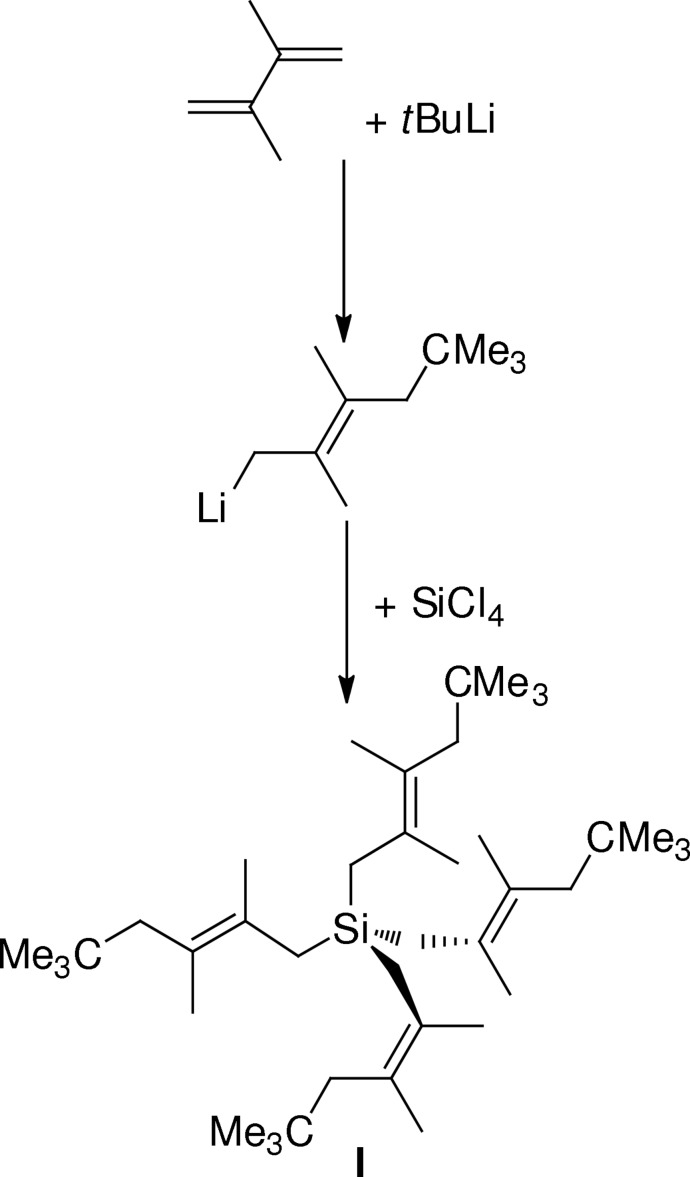
Reaction of SiCl4 with tBuLi in the presence of 2,3-dimethyl-1,3-butadiene.

**Fig. 2.**
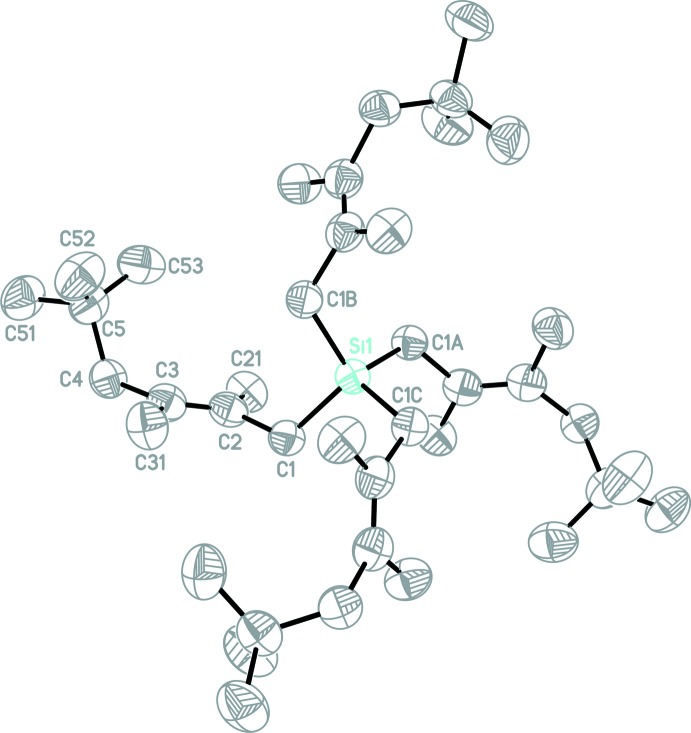
Perspective view of the title compound with displacement ellipsoids drawn at the 50% probability level. H atoms are omitted for clarity. Symmetry codes: (A) -x + 1, -y + 3/2, z; (B) y - 1/4, -x + 5/4, -z + 1/4; (C) -y + 5/4, x + 1/4, -z + 1/4.

**Fig. 3.**
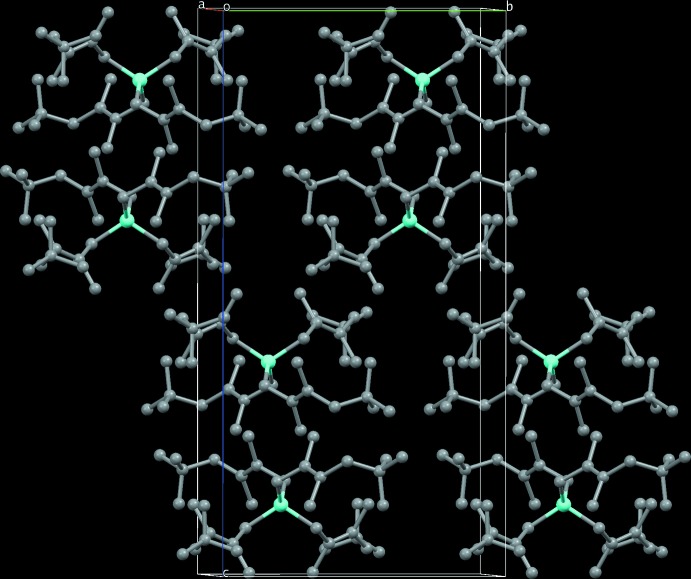
Packing diagram of the title compound; hydrogen atoms are omitted for clarity. The view direction is approximately down the a axis.

## supplementary crystallographic information

### 1. Comment

Very recently, we have investigated the reaction of Si_2_Cl_6_ with N*R*_3_ (*R* = Me, Et) and we have been able to show that amine-complexed dichlorosilylenes represent the key intermediates in this disproportionation reaction (Meyer-Wegner *et al.*, 2011). In the course of these studies we isolated along with SiCl_4_ the [4 + 1] cycloadduct of dichlorosilylene SiCl_2_ with 2,3-dimethyl-1,3-butadiene (DMB) in good yield upon treatment of Si_2_Cl_6_ with catalytic amounts of NMe_3_ or NMe_2_Et in neat DMB (Meyer-Wegner *et al.*, 2014). In this study we report on the reaction of Si_2_Cl_6_ with catalytic amounts of NMe_2_Et in the presence of DMB. Subsequent treatment of this reaction mixture with *t*BuLi yielded the title compound (I) which could be isolated (Fig. 1).

In the title compound (Fig. 2), the Si centre is located on a special position of site symmtry 4. Thus, there is just a quarter of a molecule in the asymmetric unit. The C=C double bonds show a *trans* configuration. It is interesting to note, that the Si centre and the *t*-butyl group are located on the same side of the plane formed by the C=C double bond and its four substituents. The crystal packing (Fig. 3) shows no short contacts between the molecules and despite the fact that the crystal density is rather low [0.980 g/cm^3^], there are no significant voids in the structure.

### 2. Experimental

To Si_2_Cl_6_ (0.3 ml, 0.47 g, 1.74 mmol) in DMB (1.2 ml, 0.86 g, 10 mmol) a catalytic amount of NMe_2_Et (0.012 g, 0.2 mmol) was added. Subsequent treatment of this mixture with a solution of *t*BuLi (16 mmol) in 10 ml of pentane yielded a colorless precipitate. After filtration all volatiles were removed *in vacuo*. The obtained residue was dissolved in benzene. This solution was stored at ambient temperature for a period of two days yielding single crystals of the title compound.

### 3. Refinement

H atoms were geometrically positioned and refined using a riding model with fixed individual displacement parameters [*U*(H) = 1.2 *U*_eq_(C) or *U*(H) = 1.5 *U*_eq_(C_methyl_)] and with C_methyl_—H = 0.98 Å and C_methylene_—H = 0.99 Å. The methyl groups attached to the double bond were allowed to rotate but not to tip.

### Crystal data

**Table:**

C_40_H_76_Si	*D*_x_ = 0.980 Mg m^−^^3^
*M**_r_* = 585.10	Mo *K*α radiation, λ = 0.71073 Å
Tetragonal, *I*4_1_/*a*	Cell parameters from 4399 reflections
Hall symbol: -I 4ad	θ = 3.7–25.9°
*a* = 12.5780 (11) Å	µ = 0.08 mm^−^^1^
*c* = 25.053 (3) Å	*T* = 173 K
*V* = 3963.5 (7) Å^3^	Block, colourless
*Z* = 4	0.32 × 0.28 × 0.16 mm
*F*(000) = 1320	

### Data collection

**Table:**

Stoe IPDS-II two-circle diffractometer	1742 independent reflections
Radiation source: fine-focus sealed tube	1301 reflections with *I* > 2σ(*I*)
Graphite monochromator	*R*_int_ = 0.045
ω scans	θ_max_ = 25.0°, θ_min_ = 3.6°
Absorption correction: multi-scan (*MULABS*; Spek, 2009; Blessing, 1995)	*h* = −8→14
*T*_min_ = 0.963, *T*_max_ = 0.988	*k* = −14→14
5117 measured reflections	*l* = −25→29

### Refinement

**Table:**

Refinement on *F*^2^	Primary atom site location: structure-invariant direct methods
Least-squares matrix: full	Secondary atom site location: difference Fourier map
*R*[*F*^2^ > 2σ(*F*^2^)] = 0.051	Hydrogen site location: inferred from neighbouring sites
*wR*(*F*^2^) = 0.136	H-atom parameters constrained
*S* = 1.08	*w* = 1/[σ^2^(*F*_o_^2^) + (0.0654*P*)^2^ + 0.753*P*] where *P* = (*F*_o_^2^ + 2*F*_c_^2^)/3
1742 reflections	(Δ/σ)_max_ = 0.001
95 parameters	Δρ_max_ = 0.25 e Å^−^^3^
0 restraints	Δρ_min_ = −0.17 e Å^−^^3^

### Special details

**Table:**

Geometry. All e.s.d.'s (except the e.s.d. in the dihedral angle between two l.s. planes) are estimated using the full covariance matrix. The cell e.s.d.'s are taken into account individually in the estimation of e.s.d.'s in distances, angles and torsion angles; correlations between e.s.d.'s in cell parameters are only used when they are defined by crystal symmetry. An approximate (isotropic) treatment of cell e.s.d.'s is used for estimating e.s.d.'s involving l.s. planes.
Refinement. Refinement of *F*^2^ against ALL reflections. The weighted *R*-factor *wR* and goodness of fit *S* are based on *F*^2^, conventional *R*-factors *R* are based on *F*, with *F* set to zero for negative *F*^2^. The threshold expression of *F*^2^ > σ(*F*^2^) is used only for calculating *R*-factors(gt) *etc*. and is not relevant to the choice of reflections for refinement. *R*-factors based on *F*^2^ are statistically about twice as large as those based on *F*, and *R*- factors based on ALL data will be even larger.

### Fractional atomic coordinates and isotropic or equivalent isotropic displacement parameters (Å^2^)

**Table:**

	*x*	*y*	*z*	*U*_iso_*/*U*_eq_	
Si1	0.5000	0.7500	0.1250	0.0457 (3)	
C1	0.52419 (16)	0.62670 (16)	0.08358 (7)	0.0522 (5)	
H1A	0.5766	0.6452	0.0556	0.063*	
H1B	0.5581	0.5731	0.1070	0.063*	
C2	0.43057 (17)	0.57385 (17)	0.05637 (8)	0.0564 (5)	
C3	0.38412 (17)	0.48729 (17)	0.07519 (8)	0.0595 (6)	
C4	0.29391 (17)	0.42817 (18)	0.04703 (8)	0.0588 (5)	
H4A	0.3062	0.3510	0.0518	0.071*	
H4B	0.2996	0.4432	0.0084	0.071*	
C5	0.17836 (18)	0.45239 (19)	0.06442 (8)	0.0628 (6)	
C21	0.3961 (2)	0.62822 (18)	0.00476 (8)	0.0652 (6)	
H21A	0.4590	0.6459	−0.0165	0.098*	
H21B	0.3570	0.6935	0.0133	0.098*	
H21C	0.3500	0.5802	−0.0156	0.098*	
C31	0.4258 (2)	0.43428 (18)	0.12553 (9)	0.0695 (6)	
H31A	0.3791	0.3750	0.1353	0.104*	
H31B	0.4274	0.4862	0.1547	0.104*	
H31C	0.4979	0.4075	0.1191	0.104*	
C51	0.1061 (2)	0.3674 (2)	0.03927 (11)	0.0833 (8)	
H51A	0.1139	0.3691	0.0004	0.125*	
H51B	0.0320	0.3818	0.0488	0.125*	
H51C	0.1266	0.2970	0.0526	0.125*	
C52	0.1662 (2)	0.4487 (3)	0.12469 (10)	0.0881 (8)	
H52A	0.0924	0.4648	0.1343	0.132*	
H52B	0.2136	0.5013	0.1410	0.132*	
H52C	0.1848	0.3775	0.1376	0.132*	
C53	0.1450 (2)	0.5620 (2)	0.04480 (11)	0.0825 (7)	
H53A	0.1528	0.5654	0.0059	0.124*	
H53B	0.1902	0.6162	0.0614	0.124*	
H53C	0.0707	0.5749	0.0545	0.124*	

### Atomic displacement parameters (Å^2^)

**Table:**

	*U*^11^	*U*^22^	*U*^33^	*U*^12^	*U*^13^	*U*^23^
Si1	0.0476 (4)	0.0476 (4)	0.0418 (5)	0.000	0.000	0.000
C1	0.0517 (11)	0.0526 (11)	0.0524 (10)	0.0025 (9)	−0.0010 (8)	−0.0013 (8)
C2	0.0612 (12)	0.0571 (12)	0.0508 (11)	0.0073 (10)	0.0031 (9)	−0.0075 (9)
C3	0.0636 (13)	0.0606 (13)	0.0543 (11)	0.0055 (10)	−0.0024 (9)	−0.0053 (9)
C4	0.0644 (13)	0.0599 (12)	0.0520 (10)	−0.0025 (10)	−0.0035 (9)	−0.0116 (9)
C5	0.0613 (13)	0.0744 (15)	0.0528 (11)	−0.0074 (11)	−0.0035 (10)	−0.0120 (10)
C21	0.0841 (16)	0.0659 (14)	0.0457 (10)	0.0062 (12)	−0.0018 (10)	0.0016 (9)
C31	0.0832 (15)	0.0641 (13)	0.0613 (12)	−0.0020 (12)	−0.0165 (11)	0.0081 (11)
C51	0.0674 (15)	0.0991 (19)	0.0833 (16)	−0.0160 (14)	−0.0095 (12)	−0.0227 (14)
C52	0.0807 (17)	0.122 (2)	0.0618 (13)	−0.0206 (16)	0.0092 (12)	−0.0160 (14)
C53	0.0744 (16)	0.0910 (19)	0.0821 (16)	0.0154 (14)	−0.0009 (12)	−0.0141 (13)

### Geometric parameters (Å, º)

**Table:**

Si1—C1^i^	1.8906 (19)	C5—C51	1.538 (3)
Si1—C1^ii^	1.8906 (19)	C21—H21A	0.9800
Si1—C1	1.8907 (19)	C21—H21B	0.9800
Si1—C1^iii^	1.8907 (19)	C21—H21C	0.9800
C1—C2	1.514 (3)	C31—H31A	0.9800
C1—H1A	0.9900	C31—H31B	0.9800
C1—H1B	0.9900	C31—H31C	0.9800
C2—C3	1.323 (3)	C51—H51A	0.9800
C2—C21	1.526 (3)	C51—H51B	0.9800
C3—C31	1.520 (3)	C51—H51C	0.9800
C3—C4	1.529 (3)	C52—H52A	0.9800
C4—C5	1.548 (3)	C52—H52B	0.9800
C4—H4A	0.9900	C52—H52C	0.9800
C4—H4B	0.9900	C53—H53A	0.9800
C5—C52	1.518 (3)	C53—H53B	0.9800
C5—C53	1.522 (4)	C53—H53C	0.9800
			
C1^i^—Si1—C1^ii^	107.53 (6)	C2—C21—H21A	109.5
C1^i^—Si1—C1	113.43 (12)	C2—C21—H21B	109.5
C1^ii^—Si1—C1	107.53 (6)	H21A—C21—H21B	109.5
C1^i^—Si1—C1^iii^	107.53 (6)	C2—C21—H21C	109.5
C1^ii^—Si1—C1^iii^	113.43 (12)	H21A—C21—H21C	109.5
C1—Si1—C1^iii^	107.53 (6)	H21B—C21—H21C	109.5
C2—C1—Si1	118.82 (14)	C3—C31—H31A	109.5
C2—C1—H1A	107.6	C3—C31—H31B	109.5
Si1—C1—H1A	107.6	H31A—C31—H31B	109.5
C2—C1—H1B	107.6	C3—C31—H31C	109.5
Si1—C1—H1B	107.6	H31A—C31—H31C	109.5
H1A—C1—H1B	107.0	H31B—C31—H31C	109.5
C3—C2—C1	122.99 (19)	C5—C51—H51A	109.5
C3—C2—C21	123.0 (2)	C5—C51—H51B	109.5
C1—C2—C21	113.95 (18)	H51A—C51—H51B	109.5
C2—C3—C31	120.3 (2)	C5—C51—H51C	109.5
C2—C3—C4	124.31 (19)	H51A—C51—H51C	109.5
C31—C3—C4	115.20 (19)	H51B—C51—H51C	109.5
C3—C4—C5	118.12 (16)	C5—C52—H52A	109.5
C3—C4—H4A	107.8	C5—C52—H52B	109.5
C5—C4—H4A	107.8	H52A—C52—H52B	109.5
C3—C4—H4B	107.8	C5—C52—H52C	109.5
C5—C4—H4B	107.8	H52A—C52—H52C	109.5
H4A—C4—H4B	107.1	H52B—C52—H52C	109.5
C52—C5—C53	108.7 (2)	C5—C53—H53A	109.5
C52—C5—C51	109.1 (2)	C5—C53—H53B	109.5
C53—C5—C51	109.6 (2)	H53A—C53—H53B	109.5
C52—C5—C4	111.62 (19)	C5—C53—H53C	109.5
C53—C5—C4	110.24 (19)	H53A—C53—H53C	109.5
C51—C5—C4	107.60 (18)	H53B—C53—H53C	109.5
			
C1^i^—Si1—C1—C2	−75.32 (15)	C1—C2—C3—C4	−176.17 (18)
C1^ii^—Si1—C1—C2	165.92 (18)	C21—C2—C3—C4	2.6 (3)
C1^iii^—Si1—C1—C2	43.44 (12)	C2—C3—C4—C5	−96.9 (3)
Si1—C1—C2—C3	−101.5 (2)	C31—C3—C4—C5	88.3 (2)
Si1—C1—C2—C21	79.6 (2)	C3—C4—C5—C52	−48.5 (3)
C1—C2—C3—C31	−1.6 (3)	C3—C4—C5—C53	72.4 (2)
C21—C2—C3—C31	177.2 (2)	C3—C4—C5—C51	−168.2 (2)

Symmetry codes: (i) −*x*+1, −*y*+3/2, *z*; (ii) −*y*+5/4, *x*+1/4, −*z*+1/4; (iii) *y*−1/4, −*x*+5/4, −*z*+1/4.
